# Different surgical techniques that influenced internal hernia prevalence rate after laparoscopic roux-en-Y gastric bypass: a retrospective analysis of 331 cases

**DOI:** 10.1186/s12893-020-00713-y

**Published:** 2020-03-16

**Authors:** Jingge Yang, Bingsheng Guan, Shifang Huang, Juzheng Peng, Tsz Hong Chong, Cunchuan Wang, Tsz Kin Mak

**Affiliations:** 1 Department of Gastrointestinal Surgery, First Affiliated Hospital of Jinan University, Guangzhou, 510630 China; 2 Department of Intensive Care Unit, First Affiliated Hospital of Jinan University, Guangzhou, 510630 China

**Keywords:** Laparoscopic roux-en-Y gastric bypass, Mesenteric defects, Internal hernia

## Abstract

**Background:**

Internal hernia (IH) is a serious complication following laparoscopic Roux-en-Y gastric bypass (LRYGB), and closure of mesenteric defect has been recommended to reduce this complication. But what kind of material about suture and how to close the mesenteric defects were still controversial. The main aim of this study was to compare the incidence rate of internal hernia after LRYGB between patients with different surgical techniques.

**Method:**

Three hundred and thirty-one patients underwent LRYGB between June 2004 and December 2017 in one single institute were retrospective analysed. The IH rate was evaluated according to different surgical methods and surgical materials before and 12 months after LRYGB.

**Results:**

All the cases were subdivided into three groups based on the suturing method, Roux limb position, and Suture material. The mean follow up time was 36 ± 12 months, and the total incident rate of IH was 1.8% (*n = 6*). In the six IH cases, the duration of IH occurred time ranged from 1 month to 36 months postoperatively, and for the IH sites, one for intestinal defect, three for transverse mesocolon defect and two Peterson defect respectively. There was a significant difference about IH rate between interrupted suture and running suture groups (*p = 0.011*), and there were no significant differences between the other two groups.

**Conclusion:**

Compare with interrupted suture, running suture may prevent IH after LRYGB. Patient’s gender, age, body mass index(BMI), glycometabolism condition, and Roux limb position and suture material had no effects on the IH prevalence after LRYGB.

## Background

The rising prevalence of overweight and obesity has been defined as a global pandemic [1]. Bariatric surgery is considered as the most efficient treatment for morbid obesity [2]. And one of the most commonly performed procedures was laparoscopic Roux-en-Y gastric bypass (LRYGB) [2]. In America, nearly 40,000 LRYGBs were performed in 2016 [3]. Due to this high volume, even a minor surgical technique difference may affect a lot of patients.

In spite of the increasing number of LRYGB procedures in China [4], some of postoperative complications are easy to be overlooked, including the complications that may cause severe consequences like internal hernia (IH). As the main cause of postoperative bowel obstruction, IH has been reported with the prevalence rate of 0.2–9% [5–9]. To be more specific, the IH rate after LRYGB is around 2.5%, of which 69% occurred in the transverse mesocolon defect, 18% occurred in the Petersen defect, and 13% occurred in the jejunum mesentery defect [10].

Therefore, the incidence of IH should be decreased by closing mesenteric defects [11], which can reduce the rate of small bowel obstruction from 10 to 5.5% [12]. However, related studies on this topic are still quite rare in Asia, especially in China. The aim of this study was to examine the effect of different surgical techniques on IH after LRYGB.

## Methods

A retrospective analysis of 331 cases underwent LRYGB between June 2004 and Dec 2017 in the Department of metabolic and obesity surgery of the First Affiliated Hospital of Jinan University (Guangzhou, China) were conducted. All procedures were performed by the same surgical team. Inclusion criteria for the study were Chinese patients aged from 18 to 65 years, and BMI ≥35 kg/m^2^ or a BMI ≥27.5 kg/m^2^ with type 2 diabetes or metabolic syndrome that cannot be controlled by lifestyle alternations and medical treatment. Exclusion criteria were patients who had previously bariatric procedures, abdominal surgery, mental diseases, and severe chronic disease. Three hundred thirty-one patients undergoing the surgical operation of LRYGB were evaluated by nutritionist, psychiatrist, and anesthetist preoperatively for their nutritional status, mental state, and cardio-pulmonary functions respectively. All patients have clear fluids up to 4 h and solids up to 8 h prior to induction of anaesthesia. And the anaesthesia were operated by experienced anesthetist. Bi-level positive airway pressure was used on patients with Obstructive Sleep Apnea preoperatively and all patients postoperatively. Clear liquid meal was initiated at first day after surgery, semiliquid meal was taken after upper gastrointestinal tract radiography. Gastric mucosal protective drug and proton pump inhibitor were administrated immediately after surgery and last for 2 months. They were advised to take calcium tablet and multivitamin supplementations for life. All patients were discharged 5 days after surgery.

Data recorded included gender, age, height, weight, BMI, diabetes and surgical techniques, such as different suturing method, suture material and the Roux limb position. All patients were followed up at 1,3,6, 12 months and one time for each year after 12 months postoperatively regularly. And only the cases that had been followed up for more than 12 months after LRYGB has been presented in this study. Weight loss outcomes were reported as percentage of excess weight loss(%EWL). %EWL was computed by the formula as followed: [Initial weight(kg) - current weight(kg)]/[initial weight(kg) - ideal weight(kg)]X100%, where ideal weight (kg) = 25 × [height(m)]^2^. And complications have been reported during the follow-up. As a retrospective study, ethnic approval was not necessary due to the local ethics committee (Ethics Committee of the First Affiliated Hospital of Jinan University).

### Surgical technique

The LRYGB was performed through a 5-port technique with the patient supine position. The gastric pouch was designed with the volume of approximately 10–20 ml. The length of the Biliopancreatic limb was 25 cm, and the Roux limb was 150 cm in simple obesity and 175 cm in obesity with diabetes mellitus. The Roux limb was positioned through a retro-colic or ante-colic method and fixed to the transverse mesocolon with absorbable or nonabsorbent braided sutures. The anastomotic stoma between the stomach and jejunum was 1.5 cm and 6 cm between jejunum and jejunum. The jejunum mesentery defect, transverse mesocolon defect, and Peterson defect were closed by running suture or interrupted suture, and absorbable suture or non-absorbable suture. No drainage tube was used in all patients. There was no conversion to open operation, and no mortality.

### Statistical analysis

Statistical analysis was carried out using the Statistical Product and Service Solutions version 13.0 (SPSS13.0). Continuous data were presented as mean standard deviation and categorical data were expressed as percentage (%). Subgroups were compared using Chi-square or Fisher’s exact test. A *p* value < 0.05 was considered to be statistically significant.

## Results

### General information

Three hundred thirty-one patients successfully received LRYGB procedure, with186 (56.2%) were female, 145 (43.8%) were male. The mean age was 32.1 ± 11.4 years old, the mean operation time was 125 ± 15.4 min, and the mean blood loss was 8 ± 3.0 ml. The mean BMI before surgery were 43.7 ± 11.1 kg/m^2^, reduced to 27.3 ± 10.7 kg/m^2^ after 12 months, the %EWL post-operation 12 months were 82.1 ± 9.2% (Table 1).

**Table 1:** Patient demographics

	All Patients(*n* = 331)
Gender	
Female, n(%)	186 (56.2)
Male, n (%)	145 (43.8)
Mean age (years)	32.1 ± 11.4
Mean operation time(minutes)	125 ± 15.4
Mean blood loss(ml)	8 ± 3.0
Mean BMI(kg/m^2^)	
Pre-operation	43.7 ± 11.1
Post-operation 12 months	27.3 ± 10.7
Post-operation 12 months mean %EWL	82.1 ± 9.2%

*BMI* body mass index, *%EWL* percentage excess weight loss

### IH cases

Among these patients, 6 (1.8%) encountered IH after LRYGB, 4 were females and 2 were males. The age ranged from 26 to 48 years old, BMI ranged from 32.6 to 48.2 kg/m^2^. Three patients were discovered IH 1 month after surgery, 1 patient was found 3 months after surgery, and one IH occurred 17 months after surgery, and another IH occurred 36 months postoperatively. All 6 patients’ initial symptom was abdominal pain, each of them have underwent computed tomography scan and diagnosis with IH. All of the IH cases received reoperations. Four patients who occurred IH in 1 to 3 months were successfully conducted laparoscopic hernia repair, and one case occurred IH 17 months postoperatively cured by open hernia repair. Another case who had IH complicated with bile pancreatic limb perforation 36 months postoperatively received open hernia repair and 5 cm bile pancreatic limb resection. During the hernia repairing surgery, we found that 3 IH occurred at transverse mesocolon defect, 2 occurred at Peterson defect, and 1 occurred at jejunum mesentery defect. As to the herniated segment of intestine, 4 of them were common limb, 1 of them was Biliopancreatic limb and common limb (Table 2).

**Table 2:** Information of IH patients^a^

Patient	BMI(kg/m^2^) at LRYGB	IH occurred time after surgery(months)	BMI(kg/m^2^)at IH occurred time point	IH site	Herniated segment of intestine	Treatment
1	35.2	1	32	Intestinal defect	CL	Laparoscopic intestinal defect repair
2	34.8	1	31.4	TMD	CL	Laparoscopic transverse mesocolon defect repair
3	32.6	1	30.1	Peterson defect	CL	Laparoscopic Peterson defect repair
4	32.6	17	25.3	TMD	BPL and CL	Open transverse mesocolon defect repair
5	46	3	39.5	Peterson defect	CL	Laparoscopic Peterson defect repair
6	48.2	36	27.3	TMD	CL	Open transverse mesocolon defect repair and perforated biliopancreatic limb resection

*IH* internal hernia, *TMD* transverse mesocolon defect, *BMI* body mass index, *CL* Common limb, *BPL* Biliopancreatic limb

^a^4 females and 2 males with age range from 26 to 35 years old

### Subgroup of the IH

Three hundred thirty-one cases were divided into three subgroups depend on the suturing methods, Roux limb position, and suture material. According to the suturing methods, 157 cases belonged to the interrupted suture group, which had 6 cases of IH, and 174 cases belonged to the running suture group, which had no IH case. The prevalence rate of IH was higher for interrupted suture than running suture after LRYGB (*p = 0.011).* According to the relative orientation of Roux limb to transverse colon, there was no significant difference between the ante-colic group and retro-colic group. One hundred thirty cases were divided to the antecolic group, which had 3 IH case; and 201 cases were divided to the retro-colic group,, which had 3 cases of IH occurred(*p*>0. 05). According to the difference of suture materials, there were absorbable group and non-absorbable group. Two hundred fifty-three cases belonged to the absorbable group, which had 5 cases of IH; and 78 cases belonged to the non-absorbable group, which had 1 cases of IH. There did not have a significant difference between absorbable group and non-absorbable group (*p*>0. 05).

Patients characteristics such as age, gender, BMI and Glycometabolism were also documented. There are 2 IH cases among 145 male patients, compared to 4 IH cases among 186 female patients(*p*>0. 05). Among the 156 patients who had type 2 diabetes, 3 patients had IH; and there were 3 IH cases in 175 patients without diabetes(*p*>0. 05).93 patient’s age were over 40 years at LRYGB time point and 2 of them developed IH, 238 patient’s age under 40 years old and 4 of them developed IH(*p*>0. 05). Seventy-nine patient’s BMI at LRYGB time point were over 50 kg/m^2^and none of them developed IH, 252 patient’s BMI under 50 kg/m^2^and 6 of then developed IH(*p*>0. 05) (Table 3).

**Table 3:** Incidence of different surgical technique and patient characteristics

	n(IH cases)	P
Suturing method		
Interrupted group	157 (6)	/
Running group	174 (0)	0.011
Roux limb position		
Antecolic group	130 (3)	/
Retrocolic group	201 (3)	0.904
Suture material		
Absorbable group	253 (5)	/
Non-absorbable group	78 (1)	1.000
Gender		
Male	145 (2)	/
Female	186 (4)	0.915
Glycometabolism		
Type 2 diabetes	156 (3)	/
Non-diabetes	175 (3)	1.000
Age at LRYGB		
≧40(years)	93 (2)	/
<40(years)	238 (4)	1.000
BMI at LRYGB		
≧50(kg/m^2^)	79 (0)	/
<50(kg/m^2^)	252 (6)	0.342

*IH* internal hernia, *BMI* body mass index

## Discussion

The benefits of gastric bypass are not limited to long term weight loss, it’s also associated with a significant improvement of obesity-related morbidity, such as hypertension, type 2 diabetes, sleep apnoea [13]. But the IH after gastric bypass is a potentially serious complication, especially in the patients who had undergone LGYGB, which possibly had a higher IH rate than open gastric bypass [14]. And close the mesenteric defects are proved to be an effective way to prevent the occurrence of IH [12, 15]. However, there were few researches focusing on the suture methods used to close the mesenteric defects. To our knowledge, our article is the first study to compare primary closure methods of the mesenteric defects in LRYGB surgery in China.

Various surgical methods for preventing the occurrence of IH during surgery have been described in the literature, such as closure of the mesenteric defects with non-absorbable suture in a running fashion [14], and choosing an ante-colic approach rather than retro-colic approach [16]. But the effectiveness of the methods above is still uncertain. In our study, we retrospectively analyzed three different surgical method’s effect on the incidence rate of IH after LRYGB. Ante-colic approach seems to be a better way to prevent IH rather than retro-colic approach. Three mesenteric defects are created during a retro-colic Roux-en-Y gastric bypass procedure, which were transverse mesocolon, Peterson’s and jejunum mesentery defect. Ante-colic approach only creates Peterson’s and jejunum mesentery defect, without jejunum mesentery defect. Thereby, ante-colic approach will not develop transverse mesocolon hernias. According to the research conducted by K. E. Steele et. Al, ante-colic ante-gastric technique provides better exposure and easier closure of the jejunojejunostomy mesenteric defect [16]. But in our study, with meticulous closure of all potential mesenteric defects by an experienced surgeon, retro-colic technique shows no difference at the IH rate after LRYGB when compared to ante-colic approach.

In this study, we used 3–0 barb sutures produced by Johnson & Johnson as absorbable suture material, silk sutures as non-absorbable suture material. Non-absorbable suture is the most recommended suture material for closure of mesenteric defects in many studies [15, 16]. But its lack of solid evidence to support this viewpoint. Our study shows that using absorbable or non-absorbable suture material did not affect the occurrence rate of IH after RYGB. We believe that the key point to prevent IH is the adhesion after the suture instead of the material of the suture.

Running suture is usually faster and associated with a lower leakage rate [17, 18]. But it also raises concerns for a higher incidence of stricture [19]. Our study showed that the incidence of IH was significantly reduced when the mesenteric defects were closed with running fashion (3.3% in the interrupted closure group versus 0.0% in running the closure group, *p* < 0.05). Running closure technique seems to have better leakproofness to reduce the incidence of IH compared with interrupted closure fashion.

Table 2 contains information of 6 IH cases. It tells us that IH can occur at three potential sites. 3 IH cases occurred from transverse mesocolon defect, 2 IH cases occurred from Peterson defect and only 1 IH case occurred from jejunum mesentery defect. In addition,3 of them occurred 1 month after surgery, 1 of them was 17 months, the latest one was 36 months.

In our experience, the retro-colic LRYGB would change the anatomy of gastrointestinal tract, where the alimentary limb is brought up to the gastric pouch, resulting in three potential sites at which the hernia can occur. One potential site is located between the mesentery of two small bowel loops at the jejunojejunostomy mesenteric defect. The second space where an IH could occur is the Petersen space, between the mesentery of the alimentary limb and the transverse colon, mesocolon, or retroperitoneum which the small intestine may become interposed. The third is the transmesocolic space because biliary limb needed to pass through a retro-colic tunnel. Even though some reports reveal that antecolic approach has advantages like reduction of the risk of bleeding and pancreatic damage, reduction of surgical time and less potential defects in the technique [20]. In our opinion, we prefer the retro-colic and ante-gastric approach with some advantages: (1) reduction of incidence of afferent loop syndrome because of retro-colic gastrojejunostomy is a short afferent loop, also obviously beneficial to reduce gastrojejunal tension for anastomotic healing; (2) reduction the pressure of transverse colon after the gastrojejunostomy anastomosis, the incidence of postoperative constipation, small intestine and anterior abdominal wall adhesions rate is low, function of greater omentum is not affected, more reasonable in the physiological structure.

But there are still some shortcomings of our study. At first, this is a retrospective study, more evidence-based research is needed to prove it. Second, 6 cases are small sample and the statistical results are not very convincing, high quality evidence from randomized trials are needed.

## Conclusion

Compared with the interrupted suturing, running suture closure may prevent internal hernia after LRYGB. Patient’s gender, age, body mass index, glycometabolism condition, and surgical technique such as using an ante-colic or retro-colic approach and absorbable or non-absorbable sutures have no effect on the IH ratio after LRYGB, but there still need more solid evidence to support this conclusion.

## Data Availability

The datasets generated and analysed during the current study are not publicly available as the data also forms part of an ongoing study but are available from the corresponding author on reasonable request.

## Abbreviations

IH: Internal hernia
LRYGB: Laparoscopic Roux-en-Y gastric bypass
BMI: Body mass index
